# The JPC-SE *Position Statement on Asbestos*: A Long-Overdue Appeal by Epidemiologists to Ban Asbestos Worldwide and End Related Global Environmental Injustice

**DOI:** 10.1289/ehp.1306892

**Published:** 2013-05-01

**Authors:** Wael K. Al-Delaimy

**Affiliations:** Division of Global Health, Department of Family and Preventive Medicine, University of California, San Diego, La Jolla, California, E-mail: waldelaimy@ucsd.edu

Recently, the Joint Policy Committee (JPC) of the Societies of Epidemiology (SE), a consortium of national and international epidemiologic societies and organizations, released a statement calling for the global ban of asbestos use (JPC-SE 2012). This is not the first such call for an international ban (Collegium Ramazzini 2010), but it is a significant one because it is endorsed by 10 member organizations of the JPC-SE, numerous major epidemiologic and public health associations, and many epidemiologists (JPC-SE 2012). This position statement also highlights a case of global environmental injustice on a massive scale.

For decades asbestos has been known to cause lung cancer, mesothelioma, and other respiratory and cancerous conditions (LaDou et al. 2010). Asbestos exposure was the number one occupational health problem until its use was banned in most of the developing world (International Ban Asbestos Secretariat 2012). As a legacy of past asbestos use, the number of cases of asbestos-related diseases continues to climb every year across Canada and in many other industrialized countries. Consequently, the countries that used asbestos in the past, such as Canada, the United States, Australia, and countries throughout Europe, have either adopted a legal ban or have virtually ceased using asbestos altogether.

The science regarding the harmfulness of asbestos is consistent. Therefore, international organizations such as the World Health Organization (WHO), the World Federation of Public Health Associations, the International Commission on Occupational Health, the International Social Security Association, the International Trade Union Confederation, and the World Bank are unequivocally against the use of asbestos and are also alarmed by its increased use in low- and middle-income countries. An estimated 107,000 people die each year from occupational exposure to asbestos, and > 125 million are exposed to it (WHO 2010). Although asbestos use has been banned in the mostly high-income countries because of its harmfulness, asbestos use is increasing in middle- and low-income countries because of the effective lobbying of the asbestos industry to prevent policies that ban asbestos use; there is also a profound absence of education and awareness about asbestos’ harms in the countries using it the most.

This is quite analogous to the public health problem of tobacco use: Tobacco is being restricted and controlled in the more-developed countries, resulting in declining trends of smoking, but there has been a rapid increase in smoking prevalence in the less-developed countries (Doku 2010; WHO 2000). Much like the tobacco industry, the asbestos industry hires consultants to promote scientific arguments in its favor and to manufacture doubt (Holmes 2013), and it attacks and threatens with law suits those advocates and scientists who speak out against the hazards of asbestos (Morris and Soares 2011). Recently, the journal *Inhalation Toxicology* issued an apology for publishing four articles that were commissioned by the asbestos industry and authored by consultants who serve as expert witnesses for the industry and use the articles in their litigations (Inhalation Toxicology 2012). A court deposition regarding the research published in those articles indicated that > $850,000 was paid to one of the consultants and > $7 million dollars was provided to the company that commissioned the studies (Holm 2011).

In addition to its strategies for publication of favorable articles and litigation against legal bans of asbestos, the asbestos industry has established markets in countries that have inadequate legislation and weak public health programs and environmental organizations, enabling the sale of asbestos products. One of the most extreme examples of such global injustice is Canada, which discouraged the use of asbestos-containing products in Canada but allowed the export of these same products until September 2012 (JPC-ES 2012). Canada had also been a major global producer of chrysotile asbestos. This presents a double standard: Legislators actively protect their citizens from asbestos-containing products but do not ban the exportation of these deadly products, as if citizens from the less-developed countries are second-class global citizens.

In this age of globalization and access to information, the world has become a global village. Information can no longer be withheld from the lower-income countries and their people, and education can be easily disseminated online. There is also a general trend to address public health problems that affect large populations, regardless of national borders. These changes explain why the science of global health has recently evolved so rapidly as a form of global justice.

The economic downturn that affected the entire world, the spread of pandemic infectious diseases such as SARS (severe acute respiratory syndrome), and the United Nations declaration on noncommunicable diseases are examples of why the call for a global ban on asbestos use has greater significance now than similar calls > 14 years ago (Collegium Ramazzini 1999). The personal costs to millions of people who eventually become ill or die as a result of increasing asbestos use, as well as the cost of health care that they need, can harm emerging economies of countries such as China, Brazil, and India, which, in turn, affects the global economy as a whole.

Therefore, the JPC-SE’s position statement calling for the global ban of asbestos (JPC-SE 2012), along with the innumerable epidemiologists who have rapidly risen to support it, is timely and speaks to the injustice being inflicted by mostly high-income countries and their industries against poor and unaware populations in countries where asbestos is heavily used. This practice is simply unethical, and to be silent about it is unacceptable.

The JPC-SE asbestos statement received wide media coverage because of the scientific excellence of the societies that supported it and because of the clear message that there is no safe level of exposure to any kind of asbestos. According to the most recent International Agency for Research on Cancer (IARC) Monograph on asbestos (IARC 2009), “there is sufficient evidence in humans for the carcinogenicity of all forms of asbestos (chrysotile, crocidolite, amosite, tremolite, actinolite, and anthophyllite).” Shortly after the release of the JPC-SE position statement, the Canadian government declared that it will no longer oppose adding chrysotile asbestos to the Rotterdam Convention’s list of hazardous substances (the Rotterdam Convention is held every 2 years to deliberate on the designation of hazardous substances as recommended by its scientific review panel) (Dooley 2012; Ruff 2012), as they have in past years. The Canadian government also promised financial aid to the mining communities to promote economic activities other than asbestos mining (CBC News 2012). However, the Russian government has vested interest in their asbestos industry and is reportedly relying on an ethically controversial research collaboration to veto the ban at the convention (Holmes 2013).

Public health advocacy by environmental epidemiologists and other epidemiologists, as well as scientists and public health professionals in general, is needed to bring legitimacy and accuracy to campaigns on major public health issues such as asbestos (Weiss 2012). Public health professionals need to focus on the interests of the public over any other interest. It is, therefore, not enough for epidemiologists to publish papers in scientific journals; they must also make the effort to make policy content and information of public interest both accessible and usable by the general public.
